# The potential modulating role of thyroid cysts on the association between anti–thyroid peroxidase antibodies and subclinical hypothyroidism

**DOI:** 10.1186/s40101-026-00443-9

**Published:** 2026-08-08

**Authors:** Yuji Shimizu, Nagisa Sasaki, Asuka Oyama, Yuko Noguchi, Mutsumi Matsuu-Matsuyama, Koichiro Hamada, Shin-Ya Kawashiri, Hirotomo Yamanashi, Seiko Nakamichi, Yasuhiro Nagata, Takahiro Maeda, Naomi Hayashida

**Affiliations:** 1 Department of General Medicine, Nagasaki University Graduate School of Biomedical Sciences, Nagasaki, 852-8501 Japan; 2 Epidemiology Section, Division of Public Health, Osaka Institute of Public Health, Osaka, 537-0025 Japan; 3 Department of Community Medicine, Nagasaki University Graduate School of Biomedical Sciences, Nagasaki, 852-8523 Japan; 4 Department of Health Society and Statistics, Atomic Bomb Disease Institute, Nagasaki University, Nagasaki, 852-8523 Japan; 5 Leading Medical Research Core Unit, Nagasaki University Graduate School of Biomedical Sciences, Nagasaki, 852-8523 Japan; 6 Nagasaki University Health Center, Nagasaki, 852-8521 Japan

**Keywords:** Anti-thyroid peroxidase antibody, General population, Subclinical hypothyroidism, Thyroid cysts

## Abstract

**Background:**

In our previous study, the absence of thyroid cysts was identified as an indicator of thyroid functional strain associated with thyroid peroxidase antibody (TPO-Ab) positivity. In individuals under this continuous functional load, higher levels of thyroid-stimulating hormone are required to maintain serum thyroid hormone concentrations within the normal range, potentially leading to subclinical hypothyroidism (SCH).

However, no study has examined whether the association between TPO-Ab positivity and SCH differs according to thyroid cyst status.

**Method:**

We conducted a cross-sectional study of 1432 euthyroid Japanese participants whose free triiodothyronine (T3) and free thyroxine levels were within normal ranges. Thyroid cysts were defined as cystic lesions with a maximum diameter ≥ 2.0 mm and no solid components.

**Results:**

Among the study population, 964 participants had no thyroid cysts, 267 were TPO-Ab positive, and 80 had SCH. A significant positive association between TPO-Ab positivity and SCH was observed only in participants without thyroid cysts. After adjustment for potential confounders—including sex, age, free T3, body mass index, atherosclerosis, and hypertension—the adjusted odds ratios (95% confidence intervals) were 0.27 (0.04, 2.02) and 2.10 (1.17, 3.77) for participants with and without thyroid cysts, respectively.

**Conclusion:**

Among participants without thyroid cysts, TPO-Ab positivity was significantly associated with SCH, whereas no significant association was observed among those with thyroid cysts likely due to the small sample size. Reflecting a significant interaction between these factors, thyroid cyst status may modulate the association between TPO-Ab positivity and SCH, although further large-scale investigation is warranted.

## Introduction

Thyroid peroxidase (TPO), an enzyme expressed in thyroid follicular cells, plays a central role in thyroid hormone synthesis [1]. TPO catalyzes the oxidation of iodine, iodination of tyrosine residues in thyroglobulin, and coupling reactions that generate triiodothyronine (T3) and thyroxine (T4). Autoantibodies against TPO (TPO-Ab) are thought to exert cytotoxic effects on thyroid cells [2], thereby reducing the hormone-producing capacity per unit of thyroid tissue.

Abnormally elevated TPO-Ab levels are often observed in patients with autoimmune thyroiditis [3]. Even among individuals with normal thyroid function, those with TPO-Ab positivity may experience latent thyroid functional strain. A previous cross-sectional study of individuals with free T3 and free T4 levels within normal ranges reported a positive association between TPO-Ab positivity and thyroid-stimulating hormone (TSH) [4]. This association remained significant, even when the analysis was restricted to participants with normal TSH levels.

These findings suggest that individuals who are TPO-Ab positive may require higher TSH stimulation to maintain normal thyroid hormone concentrations, likely due to increased functional strain, compared with those who are TPO-Ab negative. Elevated TSH levels in the presence of normal free T3 and free T4 concentrations are generally defined as subclinical hypothyroidism (SCH).

Previously, we conducted a cross-sectional study of general Japanese individuals aged 40–74 years with normal thyroid function—defined as free T3 and free T4 levels within normal range—and found an inverse association between TPO-Ab titers and thyroid cysts, defined as cystic lesions with a maximum diameter ≥ 2.0 mm and no solid components [5]. The absence of thyroid cysts may reflect a state of latent cellular stress within the thyroid, potentially driven by the cytotoxic properties of TPO-Ab [2] as it targets TPO expressed on follicular cells.

Therefore, in euthyroid individuals, TPO-Ab has been reported to be positively associated with TSH [4] and inversely associated with thyroid cysts [5]. Since elevated TSH with normal-range thyroid hormones is regarded as SCH, the positive association between TPO-Ab and SCH may be observed in individuals without thyroid cysts, but not in those with thyroid cysts.

This study aims to evaluate the specific association between TPO-Ab positivity and SCH according to thyroid cyst status.

## Methods

The methods used in the present study, including assessment of thyroid function, have been described elsewhere [5–8].

### Study population

This study was approved by the Ethics Committee of the Nagasaki University Graduate School of Biomedical Sciences (project registration number: 14051404–13). Informed consent was obtained from all the participants. Written informed consent forms were used to ensure that the participants understood the objectives of the study.

All procedures involving human participants were performed in accordance with the ethical standards of the Institutional Research Committee and the 1964 Declaration of Helsinki and its later amendments.

This cross-sectional study enrolled 1,883 Japanese individuals aged 40–74 years from the town of Saza in western Japan. As recommended by the Japanese government, these individuals underwent annual medical checkups in 2014.

To minimize the influence of thyroid disease, individuals were excluded if they met any of the following criteria: a history of thyroid disease (*n* = 60); missing thyroid function data, including TSH, free T3, or free T4 levels (*n* = 17); or free T3 and/or free T4 levels outside the normal range (*n* = 77). Additionally, participants without TPO-Ab data (*n* = 295) were excluded. Individuals without carotid intima–media thickness (CIMT) data (*n* = 1) or body mass index (BMI) data (*n *= 1) were also excluded. A total of 1432 participants with a mean age of 60.8 years (standard deviation [SD] 9.0 years; range 40–74 years) were included in the final analysis.

### Data collection and laboratory measurements

Trained interviewers collected information on clinical characteristics. Fasting blood samples were collected from all the participants. Serum TSH, free T3, and free T4 levels were measured using chemiluminescent immunoassays performed by LSI Medience Corporation (Tokyo, Japan). The normal ranges for free T3 (2.1–4.1 pg/mL), free T4 (1.0–1.7 ng/dL), and TSH (0.39–4.01 μIU/mL) based on this method have been described elsewhere [9]. TPO-Ab titers were measured using standard procedures in the same laboratory, and the normal range (negative) was < 16 IU/mL [9]. Therefore, TPO-Ab positivity was defined as a TPO-Ab titer ≥ 16 IU/mL. Individuals with a serum TSH concentration > 4.01 μIU/mL were defined as having SCH.

After ≥ 5 min of rest, systolic and diastolic blood pressures were measured in the sitting position using an automated device (HEM-907; Omron, Kyoto, Japan). Hypertension was defined as systolic blood pressure ≥ 140 mmHg, diastolic blood pressure ≥ 90 mmHg, or current use of antihypertensive medication.

**Fig. 1:**
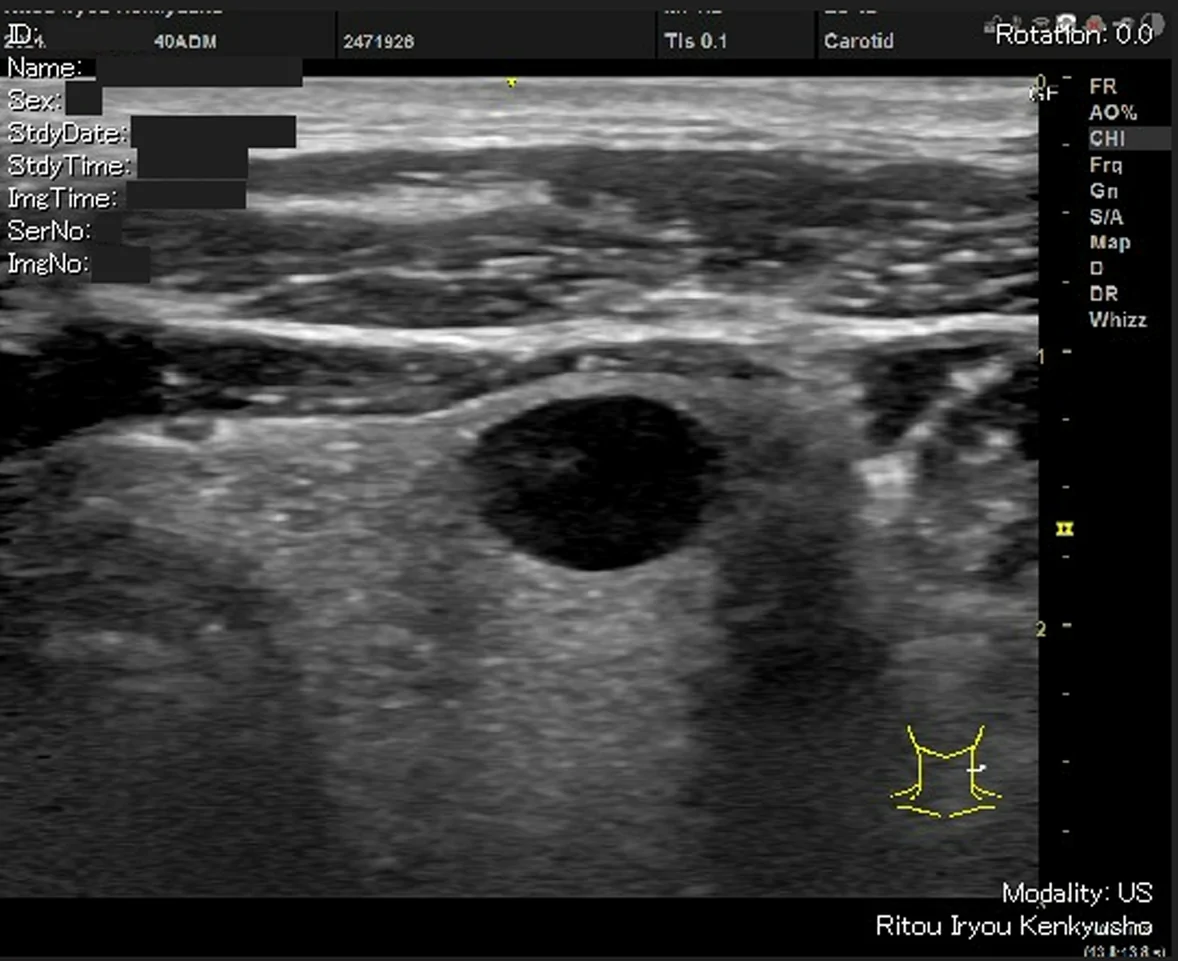
Sample of a thyroid cyst

Experienced vascular technicians measured CIMT using a LOGIQ Book XP ultrasound device with a 10 MHz transducer (GE Healthcare, Milwaukee, WI, USA). The maximum CIMT values for the left and right common carotid arteries were determined using a semi-automated digital edge-detection software (Intimascope; Media Cross, Tokyo, Japan) according to a previously established protocol [10, 11]. Plaque lesions were excluded from CIMT measurements, and the highest CIMT value was used in the analysis. Atherosclerosis was defined as CIMT ≥ 1.1 mm, in accordance with previous studies [6, 12]. In this study, a thyroid cyst was defined as a structure with a maximum diameter ≥ 2.0 mm and no solid components, consistent with previous studies [5–8]. Figure 1 shows a thyroid sample defined as a thyroid cyst in the present study.

### Statistical analyses

Thyroid cyst status–specific characteristics of the study population according to TPO-Ab positivity are presented as mean ± 1 standard deviation (SD) for continuous variables, except for TSH (age, free T3, and free T4), and as percentages for the prevalence of male sex, atherosclerosis, hypertension, high BMI, and low BMI. Because TSH values showed a skewed distribution, logarithmic transformation was performed, and the median [interquartile range] is presented.

Logistic regression models were used to calculate odds ratios (ORs) and 95% confidence intervals (CIs) to examine the association between TPO-Ab positivity and thyroid cysts and between TPO-Ab positivity and SCH. To evaluate the association between TPO-Ab positivity and SCH, stratified analyses according to thyroid cyst status were also performed. To assess the adequacy of the model in the present study population, the goodness-of-fit was evaluated using the Hosmer–Lemeshow test. Active thyroid hormones, namely, free T3, may have confounded our results. Additionally, hypertension and atherosclerosis may influence the biochemical activity of thyroid cysts [6]. Thyroid cysts have been shown to modify the association between TSH and hypertension [13]. BMI is also associated with thyroid function [14]. To counteract these influences, three models were used in this study. The first model (Model 1) was adjusted for sex and age. The second model (Model 2) was adjusted for sex, age, and free T3 levels. The third model (Model 3) was further adjusted for atherosclerosis, hypertension, high BMI, and low BMI in addition to sex, age, and free T3.

In the thyroid cyst status–specific analyses, the effect of thyroid cyst status on the association between TPO-Ab positivity and SCH was evaluated.

Additionally, using the sex-and age-adjusted model, we calculated the ORs (95% CIs) for TPO-Ab positivity in relation to the logarithmic value of TSH for the total population, for individuals without SCH, and for the total population stratified by thyroid-cyst status.

All statistical analyses were performed using SAS for Windows, version 9.4 (SAS Institute Inc., Cary, NC, USA). As in previous studies [15, 16], *p* values < 0.01 for main effects and < 0.20 for interactions were considered statistically significant.

## Results

Among the study population, 964 participants had no thyroid cysts, 267 were TPO-Ab positive, and 80 had SCH.

### Characteristics of the study population

Table 1 shows the thyroid cyst status-specific clinical characteristics of the study population according to the TPO-Ab status.

**Table 1:** Thyroid cyst status-specific characteristics of the study population according to anti-thyroid peroxidase antibody (TPO-Ab) status

	With thyroid cysts			Without thyroid cysts		
	TPO-Ab		*p*	TPO-Ab		*p*
	(−)	(+)		(−)	(+)	
No. of participants	410	58		755	209	
Men, %	30.2	36.2	0.359	41.9	33.5	0.029
Age, years	62.3 ± 7.9	64.1 ± 7.5	0.113	59.7 ± 9.5	61.2 ± 8.9	0.038
Free T3, pg/mL	3.1 ± 0.3	3.2 ± 0.3	0.573	3.2 ± 0.3	3.2 ± 0.3	0.600
Free T4, ng/dL	1.2 ± 0.1	1.2 ± 0.1	0.918	1.2 ± 0.2	1.3 ± 0.2	0.462
TSH, μIU/mL	1.6 [1.1, 2.2]^*a^	1.7 [1.1, 2.5]^*a^	0.417^*b^	1.5 [1.1, 2.2]^*a^	1.9 [1.3, 2.8]^*a^	< 0.001^*b^
Atherosclerosis, %	11.7	12.1	0.936	8.9	9.1	0.923
Hypertension, %	46.8	53.4	0.346	36.6	41.6	0.181
High BMI, %	21.7	24.1	0.677	24.5	26.8	0.499
Low BMI, %	10.2	3.4	0.097	9.3	4.3	0.021

Age, free T3, and free T4 values are presented as mean ± 1 standard deviation

^*a^Values are expressed as median [first quartile, third quartile]

^*b^Logarithmic transformation was used to evaluate* p* values. *T3* triiodothyronine, *T4* thyroxine, *TSH* thyroid-stimulating hormone, *BMI* body mass index

Among participants without thyroid cysts, those who were TPO-Ab-positive were significantly older, had a substantially lower prevalence of the male sex, low BMI, and significantly higher TSH levels than those who were TPO-Ab-negative.

### Association between TPO-Ab positivity and SCH among total participants

Table 2 shows the ORs (95% CIs) for SCH according to TPO-Ab positivity. Although not statistically significant, a positive association was observed between TPO-Ab positivity and SCH. The adjusted ORs (95% CIs) were 1.57 (0.94, 2.64) for Model 1, 1.59 (0.95, 2.68) for Model 2, and 1.54 (0.92, 2.61) for Model 3.

**Table 2:** Association between anti-thyroid peroxidase antibody (TPO-Ab) positivity and subclinical hypothyroidism

	Total participants		
	TPO-Ab		*p*
	(−)	(+)	
No. of participants	1165	267	
Participants with subclinical hypothyroidism, *n* (%)	59 (5.1)	21 (7.9)	
Model 1	Reference	1.57 (0.94, 2.64)	0.088
Model 2	Reference	1.59 (0.95, 2.68)	0.080
Model 3	Reference	1.54 (0.92, 2.61)	0.101

*Model 1* adjusted for age and sex, *Model 2* additionally adjusted for free triiodothyronine (T3), *Model 3* adjusted for age, sex, free T3, high BMI, low BMI, atherosclerosis, and hypertension, *TPO-Ab* anti-thyroid peroxidase antibody

### Association between TPO-Ab positivity and thyroid cysts among total participants

Table 3 shows the ORs (95% CIs) for thyroid cysts based on TPO-Ab positivity. TPO-Ab positivity was significantly inversely associated with thyroid cysts. The adjusted ORs (95% CIs) were 0.47 (0.34, 0.65) in all models (Models 1–3).

**Table 3:** Association between anti-thyroid peroxidase antibody (TPO-Ab) positivity and thyroid cysts

	Total participants		
	TPO-Ab		*p*
	(−)	(+)	
No. of participants	1165	267	
Participants with thyroid cysts, n (%)	410 (35.2)	58 (21.7)	
Model 1	Reference	0.47 (0.34, 0.65)	< 0.001
Model 2	Reference	0.47 (0.34, 0.65)	< 0.001
Model 3	Reference	0.47 (0.34, 0.65)	< 0.001

*Model 1* adjusted for age and sex, *Model 2* additionally adjusted for free triiodothyronine (T3), *Model 3* adjusted for age, sex, free T3, high BMI, low BMI, atherosclerosis, and hypertension, *TPO-Ab* anti-thyroid peroxidase antibody

### Association between TPO-Ab positivity and SCH according to thyroid cyst status

The thyroid cyst status-specific associations between TPO-Ab positivity and SCH are shown in Table 4. Although statistical significance was not reached among the participants with thyroid cysts, an inverse association was observed between TPO-Ab positivity and SCH. The adjusted ORs (95% CIs) were 0.27 (0.04, 2.01) for Model 1, 0.27 (0.04, 2.02) for Model 2, and 0.27 (0.04, 2.02) for Model 3. Significant positive associations were observed among participants without thyroid cysts. The corresponding ORs (95% CIs) were 2.12 (1.19, 3.77) for Model 1, 2.15 (1.21, 3.84) for Model 2, and 2.10 (1.17, 3.77) for Model 3.

**Table 4:** Association between anti-thyroid peroxidase antibody (TPO-Ab) positivity and subclinical hypothyroidism according to thyroid cyst status

	With thyroid cysts			Without thyroid cysts			*P* for interaction
	TPO-Ab		*p*	TPO-Ab		*p*	
	(−)	(+)		(−)	(+)		
No. of participants	410	58		755	209		
Participants with subclinical hypothyroidism, n (%)	24 (5.9)	1 (1.7)		35 (4.6)	20 (9.6)		
Model 1	Reference	0.27 (0.04, 2.01)	0.199	Reference	2.12 (1.19, 3.77)	0.011	0.054
Model 2	Reference	0.27 (0.04, 2.02)	0.201	Reference	2.15 (1.21, 3.84)	0.009	0.054
Model 3	Reference	0.27 (0.04, 2.02)	0.200	Reference	2.10 (1.17, 3.77)	0.013	0.053

*Model 1* adjusted for age and sex, *Model 2* additionally adjusted for free triiodothyronine (T3), *Model 3* adjusted for age, sex, free T3, high BMI, low BMI, atherosclerosis, and hypertension, *TPO-Ab* anti-thyroid peroxidase antibody

Thyroid cyst status showed a significant interaction effect on the association between TPO-Ab positivity and SCH; the *p* values for interaction were 0.054 for Models 1 and 2 and 0.053 for Model 3.

### Additional analysis

In an additional analysis, using a sex-and age-adjusted model, we evaluated the association between TPO-Ab positivity and subtle variations in TSH among all participants and among those without SCH. The sex-and age-adjusted ORs (95% CIs) for TPO-Ab positivity in relation to the logarithmic value of TSH were 1.51 (1.19, 1.90) for the total population and 1.52 (1.13, 2.03) for individuals without SCH.

We also examined thyroid‑cyst-specific ORs (95% CIs) for the association between TPO‑Ab positivity and the logarithmic value of TSH using the same adjusted model. The corresponding values were 1.61 (1.22, 2.22) for participants without thyroid cysts and 1.19 (0.74, 1.93) for those with thyroid cysts.

## Discussion

The major finding of the present study is that TPO-Ab positivity was significantly positively associated with SCH among participants without thyroid cysts, but not among those with thyroid cysts.

TPO-Ab positivity has been reported to be significantly and positively associated with TSH levels (log-transformed) within the normal range among euthyroid individuals [4]. In the present study, although a positive trend was observed between TPO-Ab positivity and SCH, statistical significance was not attained (Table 2).

However, we provide further evidence that a positive association between TPO-Ab positivity and SCH was observed only in participants without thyroid cysts (Table 4). TPO contributes to thyroid hormone synthesis [1] and TPO-Ab is an antibody against TPO. To date, conclusive evidence demonstrating that TPO-Ab directly impair TPO enzymatic function and consequently inhibits thyroid hormone synthesis is lacking. However, TPO-Ab contribute to the development of autoimmune thyroiditis [3] by mediating cytotoxic injury to thyroid follicular cells through autoimmune mechanisms directed against TPO [2]. Therefore, euthyroid individuals who are positive for TPO-Ab may harbor latent thyroid functional strain, which could ultimately challenge thyroid hormone production. Consequently, the absence of thyroid cysts may indicate this latent functional strain, as a significant inverse association was observed between TPO-Ab positivity and thyroid cysts (Table 3).

In the present study, the additional analysis demonstrated a significant positive association between TPO-Ab positivity and TSH both in the overall population and among individuals without SCH. We also observed a significant positive association between TPO-Ab positivity and TSH among participants without thyroid cysts, but not among those with cysts. These findings partially support the hypothesis that the absence of thyroid cysts reflects a state of latent functional burden on the thyroid. Individuals with reduced thyroid hormone production require elevated serum TSH levels to maintain serum thyroid hormone concentrations within the normal range, which may result in SCH. Therefore, as shown in Table 1, no significant differences in free T3 or free T4 levels were observed between TPO-Ab-negative and TPO-Ab-positive individuals, regardless of the presence or absence of thyroid cysts. However, only among individuals without thyroid cysts, TSH levels were significantly higher in those who were TPO-Ab positive compared with those who were TPO-Ab negative.

Under the influence of TPO-Ab positivity, the thyroid gland may experience latent functional strain, which is associated with the absence of thyroid cysts and SCH.

Because thyroid cyst fluid contains high concentrations of thyroglobulin, the precursor of thyroid hormones [17, 18], individuals with thyroid cysts may have an enhanced ability to meet increased hormonal demands, as these cysts function as reservoirs of thyroglobulin [6–8]. Consequently, in individuals lacking sufficient functional thyroglobulin reservoirs, TPO-Ab leads to an elevation in TSH in order to maintain thyroid hormone production.

A previous study of euthyroid individuals reported a significant inverse association between TSH and proteinuria among those with thyroid cysts, whereas a significant positive association was observed among those without cysts [7]. As thyroid hormones contribute to the prevention of chronic kidney disease [19], it is possible that, in individuals with sufficient reservoirs of thyroglobulin, higher TSH levels may reflect increased thyroid hormone activity. By contrast, in individuals with insufficient thyroglobulin reserves, higher TSH levels may indicate inadequate thyroid hormone activity. Collectively, these studies support the hypothesis that thyroid cysts function as thyroglobulin reservoirs.

Accordingly, in the present study, a significant positive association between TPO-Ab positivity and SCH was observed among participants without thyroid cysts but not among those with thyroid cysts. Although statistical significance was not reached, an inverse association between TPO-Ab positivity and SCH was observed in participants with thyroid cysts.

Physiological adaptation to impaired thyroid hormone synthesis related to TPO-Ab positivity may explain this inverse association. To maintain serum thyroid hormone levels within the normal range, participants free from latent functional burden may increase thyroid hormone synthesis independently of TSH levels.

Because TPO contributes to thyroid hormone synthesis, whereas TPO-Ab is an antibody against TPO. To clarify this mechanism, further investigation is required to evaluate the effects of TPO-Ab titers on thyroid hormone synthesis in euthyroid individuals.

To the best of our knowledge, this is the first study to demonstrate that thyroid cyst status may act as a determinant of the association between TPO-Ab positivity and SCH. Because thyroid function assessment based on blood tests performed in health checkups (free T3, free T4, TSH, and TPO-Ab) is inherently limited in its ability to evaluate true thyroid function, our findings provide new and useful insights for assessing thyroid function in health checkups, for which accurate functional evaluation is particularly challenging.

This study had several limitations. First, only the presence or absence of thyroid cysts was evaluated as described in our previous study [5–8, 13]. However, the number and size of thyroid cysts may also influence thyroid hormone activity. Further investigations that incorporate these parameters are required. Second, because of the small number of participants with SCH and thyroid cysts, statistical significance was not achieved. Therefore, further studies with larger sample sizes are warranted. Nevertheless, thyroid cyst status showed a significant interaction effect on the association between TPO-Ab positivity and SCH.

## Conclusion

In the overall population, no statistically significant association was observed between TPO-Ab positivity and SCH. However, stratified analysis revealed that a significant positive association was exclusively present among participants without thyroid cysts, whereas no such association was observed in those with thyroid cysts. Notably, a significant statistical interaction was found between TPO-Ab status and the presence of thyroid cysts. Although the limited sample size in the cystic subgroup requires cautious interpretation, these results suggest that thyroid cyst status may serve as an essential modifying factor that modulates the relationship between TPO-Ab positivity and autoimmune-related thyroid functional strain.

## Data Availability

No datasets were generated or analysed during the current study.

## Abbreviations

TPO: Thyroid peroxidase
TPO-Ab: Thyroid peroxidase antibody
SCH: Subclinical hypothyroidism
T3: Triiodothyronine
T4: Thyroxine
TSH: Thyroid-stimulating hormone
BMI: Body mass index
SD: Standard deviation
CIMT: Carotid intima–media thickness
ORs: Odds ratios
CIs: Confidence intervals
